
JOURNAL INFORMATION
==============================
NLM Title Abbreviation: ScientificWorldJournal
Journal ID: ScientificWorldJournal
NLM Journal ID: 101131163
Journal ID: TSWJ

The Scientific World Journal

ISSN: 2356-6140
EISSN: 1537-744X
Publisher: Wiley

ARTICLE INFORMATION
==============================
PMCID: PMC6009343
PMID: 29973778
DOI: 10.1100/tsw.2002.4
Article ID: 404256
Article version: 1
Subjects: Short Report

96 Well, High Throughput Translation

Allen Jason
Wallace Janette
Patel Meenal
Kudlicki Wieslaw *
Ambion Inc., 2130 Woodward Street, Austin, TX 78744-1832, USA
*Wieslaw Kudlicki: tkudlicki@ambion.com
Publication date: 2002
Electronic publication date: 2002 May 8
Volume: 2
First page: 7
Last page: 8
Copyright: Copyright © 2002 Jason Allen et al.
Copyright year: 2002
License: This is an open access article distributed under the Creative Commons Attribution License, which permits unrestricted use, distribution, and reproduction in any medium, provided the original work is properly cited.
License URL: https://creativecommons.org/licenses/by/3.0/

==============================
Advances in Gene Technology: The Genome and Beyond –
Structural Biology for Medicine (Proceedings of the 2002 Miami
*Nature Biotechnology* Winter Symposium)
*TheScientificWorld* 2002, 2(S2), 7–8
ISSN 1532-2246; DOI 10.1100/tsw.2002.4

### 96 WELL, HIGH THROUGHPUT TRANSLATION

Jason Allen, Janette Wallace, Meenal Patel, and Wieslaw Kudlicki
Ambion Inc., 2130 Woodward Street, Austin, TX 78744-1832
tkudlicki@ambion.com

**INTRODUCTION.** Ambion has developed 96 well protein expression systems using a bacterial extract for coupled transcription/translation and a eukaryotic rabbit reticulocyte lysate for translation. Each well is designed for high reproducibility and a high yield of full-length target proteins. These systems are ideal for large scale screening assays and high throughput analysis.

**RESULTS.** A high throughput bacterial coupled transcription/translation system was used to synthesize CAT protein, active green fluorescent protein (GFP), and several other eukaryotic and prokaryotic proteins. GFP activity was detected after 10 min incubation with maximum fluorescence at 45 min - measuring 52-fold over background. Other proteins effectively synthesized in this system included: IF4A, firefly luciferase, rhodanase, and Xef-1α. Proteins synthesized and detected by Western blotting include: rhodanese, RF2, L9, and L7/L12.

The high throughput retic lysate translation system also performed very well at synthesizing Xef-1α and active firefly luciferase. Nonradioactive, Western blot-based detection of newly synthesized products is also presented.

**DISCUSSION.** Two approaches have guided efforts to achieve efficient cell-free translation. The first is based on crude cell-free extract derived from *E. coli* cells. Such conventional batch systems offer several advantages, e.g., convenience of operation, easy scale-up, high throughput format and excellent reproducibility. The second approach attempts to reconstitute protein synthesis machinery from fractionated or purified components. However, lack of molecular chaperones and other folding catalysts make this system impractical for synthesis of biologically active proteins. For this reason, we utilized a complete *E. coli* system for *in vitro* synthesis of protein products and optimized this system for maximum yield and activity of products synthesized in a high throughput mode. A long-lasting, *E. coli*–based system has also been recently described by Roche (Martin et al. 2001). It allows protein expression to continue for more than 24 h. However, this system requires the use of a costly apparatus and allows only a single protein to be synthesized at a time.

Ambion's PROTEINscript-PRO 96 and Retic Lysate IVT 96 Kits allow simultaneous synthesis of 96 different proteins in a short period of time using either DNA or RNA (capped or uncapped) templates. These kits meet today's biotechnology requirements and are useful tools for large screening assays.

### REFERENCES

1. Ambion TechNotes, Volume 8, Number 5, p. 18.
2. Martin, G.A., Kawaguchi, R., Lam, Y., DeGiovanni, A., Fukushima, M., and Mutter, W. (2001) BioTechniques 31, 948–953.
